# Lower-limb coordination adaptations to shooting distance in basketball: an exploratory angular velocity-based vector coding study

**DOI:** 10.3389/fbioe.2025.1730129

**Published:** 2026-01-05

**Authors:** Ziyang Feng, Zhonghao Xie, Zhiguan Huang, Wenwei Huang, Xue Mi, Guoxing Li

**Affiliations:** 1 School of Athletic Training, Guangzhou Sport University, Guangzhou, Guangdong, China; 2 Guangdong Engineering Technology Research Center for Sports Aids, Guangzhou, China; 3 School of Sports and Health, Guangzhou Sport University, Guangzhou, China

**Keywords:** basketball, circular mixed-effect model, coordination, OpenCap, vector coding

## Abstract

**Objective:**

This exploratory study aimed to investigate how shooting distance is associated with the adaptations in lower limb inter-joint coordination of male university basketball athletes during the Loading and Jump Phases.

**Methods:**

Kinematic data from 14 university basketball athletes performing jump shots at four distances (4.6 m, 5.8 m, 7.0 m, and 8.325 m) were collected using OpenCap. Inter-joint coordination was quantified using a vector coding technique based on angular velocity data. This approach was selected to conform with biomechanical conventions for calculating angular dynamics, preserve more temporal information, and make the measure more robust to any noise that may be present in the data. Subsequently, the adaptations were analyzed using Bayesian circular mixed-effects models.

**Results:**

Analysis of the data showed a primary adaptation in the Jump Phase: as shooting distance increased, coordination showed a trend towards greater Distal (knee and ankle) Dominancy. This change was underpinned by interaction effects (95% HPD that did not contain zero) between shooting distance and movement phase in multiple lower-limb joint couplings. Alongside this primary adaptation, an asymmetrical adjustment in the bilateral Knee-Ankle coordination during the Loading Phase emerged: with increasing distance, the right side showed enhanced Distal Dominancy while the left side trended towards Proximal Dominancy.

**Conclusion:**

Based on these observed patterns, we propose two hypotheses for future confirmatory research: (1) that this trend towards greater Distal Dominancy may reflect a functional optimization of the lower limb for long-range shooting, and (2) that the observed asymmetry might be a functional adaptation to the specific demands of the shooting motion.

## 1 Introduction

In basketball, one of the world’s most popular sports, shooting stands as the sole method of scoring and is therefore considered one of the most critical technical determinants of match outcomes (Okazaki et al., 2015; Cabarkapa and Deane, 2022). Athletes are required to master shots from various distances, including short-to mid-range two-point shots and long-range three-point shots, each posing distinct biomechanical demands. Therefore, in-depth research into the shooting motion is essential for providing evidence-based training guidance to players and coaches.

Previous studies have found that body kinematics change when performing shots from different distances. Elite basketball players exhibit greater shoulder axis rotation in the transverse plane, accompanied by a significant increase in forward center of mass displacement, when making long-distance shots (Podmenik et al., 2021). Analyzing professional male players, Cabarkapa and Cabarkapa (2022) found that the preparatory phase of three-point shots exhibited greater degrees of knee and hip flexion compared to short-distance shots. Similar patterns are observed in adolescent female basketball players. To compensate for longer distances, they increase knee and elbow flexion, as well as joint angular velocities (shoulder, elbow, knee) and horizontal center of mass displacement (França et al., 2022). While these studies highlight adjustments in individual joint kinematics, previous research has not extensively investigated the specific effects of shooting distance on the inter-joint coordination strategies of the lower limbs. In practice, the motor control system does not regulate each joint or muscle individually; instead, it leverages compensatory relationships among various components to maintain performance stability and movement flexibility (Latash et al., 2007). Therefore, an analysis of the lower limb inter-joint coordination during shots from different distances is key to revealing how the motor system adapts to changes in distance.

This concept of coordination is characterized as the functional relationship of multiple elements (such as muscles and joint motions) to achieve a shared objective in a motor task (Kimura et al., 2021). Efficient coordination is a hallmark of the high-level skills exhibited by elite athletes. For example, in racewalking, elite and international-level athletes exhibit lower variability in pelvis-hip coordination during the early stance and in pelvis-ankle coordination during the propulsive phase compared to national-level athletes, indicating more stable and efficient coordination patterns (Cazzola et al., 2016). Conversely, in swimming, elite athletes generate effective coordination patterns through flexible adaptations under task constraints (Seifert et al., 2014). These contrasting examples highlight that coordination is a dynamic solution to varying constraints. As external constraints change, athletes' movement coordination adapts to meet the new functional demands (Davids et al., 2008; Shafizadeh et al., 2019). Similarly, shooting distance acts as a critical external constraint that influences movement coordination. Taken together, these findings highlight that high-level motor performance does not stem from the repetition of a single movement pattern, but rather from an athlete’s ability to flexibly organize various body parts through functional coordination strategies to achieve a goal (Bartlett et al., 2007; Davids et al., 2003).

Vector coding is a method for quantifying coordination. The conventional approach evaluates the consistency of inter-joint coordination by acquiring angular data from two joints to create an angle-angle plot and then compute the uniformity of the coupling angle over several cycles (Tepavac and Field-Fote, 2001). Building on this framework, Chang et al. (2008) extended the method by proposing that coordination could be categorized into four distinct patterns: In-Phase, Anti-Phase, Proximal Dominancy, and Distal Dominancy. However, the methodological basis for these classifications—relying on coupling angles derived from joint angle data—has faced criticism. Stock et al. (2022) contended that relying on joint angles disregards dynamic information, is susceptible to noise, and fails to preserve temporal data, suggesting that using angular velocity would enhance the robustness of the findings. Accordingly, this study employed vector coding based on angular velocity data. This approach was chosen to conform with biomechanical conventions by accounting for the fact that the angular velocity of one Euler angle component is affected by the angular positions and dynamics of other Euler angle components, thereby accurately representing three-dimensional angular dynamics (Stock et al., 2022). Additionally, by directly utilizing angular velocity data, this method preserves temporal data by automatically accounting for temporal differences between movement cycles, thus retaining information both within and between cycles (Stock et al., 2022). Finally, this method offers superior robustness to measurement noise compared to position-based methods (Stock et al., 2022).

How lower limb inter-joint coordination is reorganized during basketball jump shots to meet the demands of increased distance remains unclear. Accordingly, this study aimed to address two primary objectives: (1) To characterize how lower-limb inter-joint coordination strategies adapt across increasing shooting distances during the Loading and Jump phases; and (2) To specifically investigate whether these adaptations manifest as a systematic change in Proximal-Distal Dominancy. Recent neuromuscular evidence has shown that, compared to short-distance shooting, long-distance shooting requires a higher synergistic contribution from knee and ankle extensor muscles, particularly during the shot’s release phases (Fan et al., 2024). Therefore, adopting an exploratory study guided by this neuromuscular premise, and considering that the Jump Phase is responsible for the final propulsion of the body, we placed a particular focus on identifying potential change in coordination dominancy during this period as a key adaptive strategy.

## 2 Materials and methods

### 2.1 Participant

Fourteen male university basketball athletes (mean ± SD age: 20.43 ± 1.40 years, height: 1.83 ± 0.05 m, weight: 80.04 ± 9.05 kg) from Guangzhou Sport University were recruited for this study.

The inclusion criteria were as follows: (1) possessed a minimum of 7 years of competitive training experience; (2) classified as National Level II Basketball Athletes or above, with verified participation in provincial or national basketball leagues; (3) right-hand dominant athletes; and (4) reported no musculoskeletal impairments within the past 6 months.

The study was approved by the Ethics Committee of Guangzhou Sport University (Approval No. 2025LCLL-083), and all participants provided written informed consent prior to participation.

### 2.2 Experimental procedures

The experiment was conducted at the basketball gymnasium of Guangzhou Sport University. One half-court was used for equipment setup while participants performed a 10- to 20-min warm-up, including dynamic stretching and shooting practice, on the other. Following the official OpenCap recommendations (Uhlrich et al., 2023), a checkerboard was placed vertically on the shooting spot, facing the basket. Two iPhones (iPhone 13 and iPhone 15), each mounted on a 1.5 m tripod, were positioned 3 m in front of the checkerboard. They were placed symmetrically on the left and right sides, each angled approximately 30° toward the board’s center. After ensuring the checkerboard was centered in each phone’s view, camera calibration was performed, followed by the acquisition of a neutral-stance static trial. All data were collected using the default “Advanced Settings” (Model: LaiUhlrich2022, Pose model: HRNet, Marker Augmenter Model: LSTM, with videos recorded at 60 fps).

**FIGURE 1:**
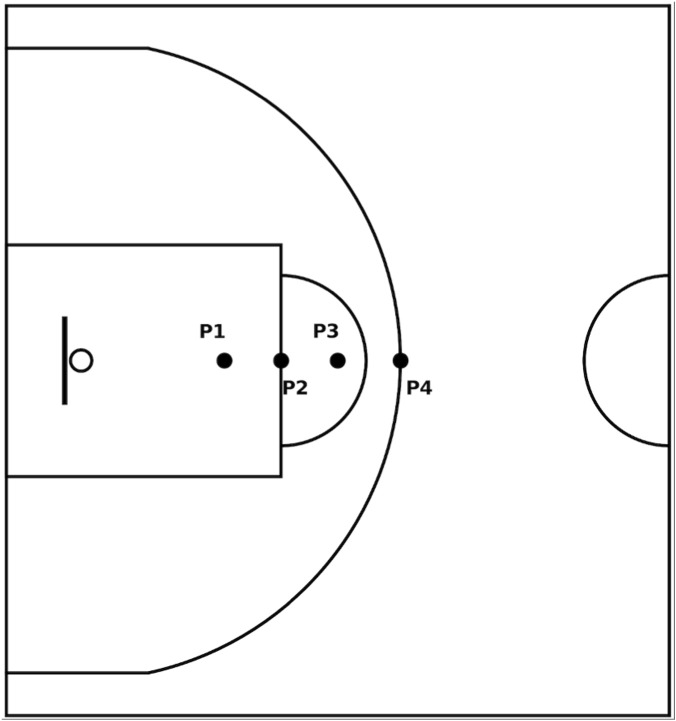
Distribution of the shooting distances. The distance of P1 from the midpoint of the baseline is 4.6 m; P2 is 5.8 m; P3 is 7.0 m; and P4 is 8.325 m.

Once the model generation was complete, the data acquisition process commenced. Participants performed shots from a total of four locations (Figure 1), which, using the midpoint of the court’s baseline as the origin, were set at perpendicular distances of 4.6 m (P1), 5.8 m (P2, free-throw line), 7.0 m (P3), and 8.325 m (P4, three-point line at the top of the arc). To minimize potential order effects, the sequence of shooting distances was randomized for each participant using an online randomization tool (www.random.org).

For each trial, participants started from an upright standing position, holding the ball at their chest with two hands, and initiated the shot following a given signal. At each location, participants performed trials until four successful shots were recorded, with a 30-s rest period between each trial to minimize fatigue. A trial was deemed successful only if the shot was made, the model generated data without error, and the participant landed in a stable, two-footed stance; otherwise, the trial was discarded and repeated. This entire process, starting from camera calibration, was repeated for each of the four shooting distances. The experimental session for each participant lasted approximately 1 hour.

### 2.3 Data analysis

All models and kinematic data generated by OpenCap were processed in OpenSim 4.5. For each trial, the corresponding inverse kinematics file was loaded into the model. Subsequently, the “Analyze” tool was utilized to compute the angular velocity and center of mass displacement. All data were then filtered using a fourth-order, low-pass Butterworth filter with a zero-phase lag at a cutoff frequency of 8 Hz, which was determined based on a residual analysis (Thomas et al., 2023).

**FIGURE 2:**
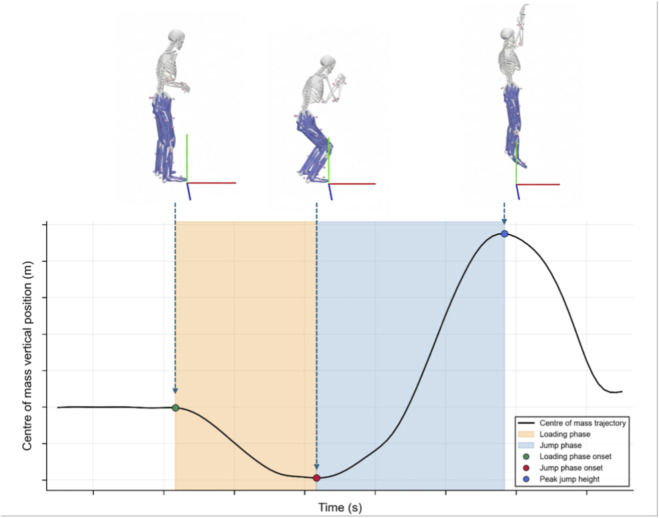
An illustrative diagram defining the phases of the jump shot.

The Global Coordinate System (GCS) in OpenCap was defined with the X-axis as anterior-posterior, the Y-axis as vertical, and the Z-axis as the medial-lateral direction. Based on the vertical displacement of the center of mass (COM), the shooting motion was divided into two distinct phases (Figure 2): 1) a Loading Phase, from the initial static position to the COM’s lowest point, and 2) a Jump Phase, from the COM’s lowest point to its highest point. Following this phase division, the sagittal plane angular velocity data for the bilateral hip, knee, and ankle joints were extracted for each phase and subsequently time-normalized to 100% within each phase for further analysis.

The inter-joint coordination for the lower limbs was quantified using the Modified Vector Coding method based on angular velocity data (Stock et al., 2022). This involved generating angular velocity-angular velocity plots for the right and left Hip-Knee, Hip-Ankle, and Knee-Ankle joint couplings. For this analysis, we defined motion direction as positive for hip and knee flexion and ankle dorsiflexion, while their counterparts—hip and knee extension and ankle plantarflexion—were defined as negative. The coupling angle for each pair was then calculated using the following formula (Equation 1) (Stock et al., 2022), where $\omega_{y}$ represents the angular velocity of the proximal joint (y-axis) and $\omega_{x}$ represents the angular velocity of the distal joint (x-axis).

$$\gamma =\tan^{-1}\left(\frac{\omega_{y}}{\omega_{x}}\right)$$ (1)

For the definition of coordination patterns, we adopted the method proposed by Needham et al. (2015), which divides the coordination pattern into four quadrants: In-phase Proximal Dominancy, In-phase Distal Dominancy, Anti-phase Proximal Dominancy, and Anti-phase Distal Dominancy (Table 1). In-Phase refers to the rotation of two segments in the same direction, whereas Anti-Phase refers to the rotation of two segments in opposite directions (Needham et al., 2020).

**TABLE 1:** Definition of coordination patterns based on coupling angle intervals.

Coordination pattern	Coordination angle bin definition
In-phase distal dominancy	$0^{\circ }\leq \gamma <45^{\circ },180^{\circ }\leq \gamma <225{}^{\circ}$
In-phase proximal dominancy	45°<γ≤90°,225°<γ≤270°
Anti-phase distal dominancy	90°≤γ<135°,270°≤γ<315°
Anti-phase proximal dominancy	135°<γ≤180°,315°<γ≤360°

**FIGURE 3:**
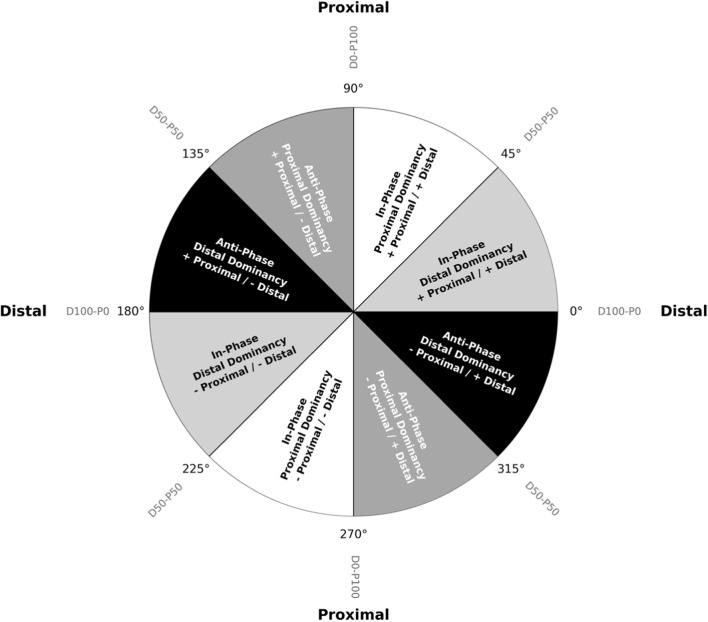
This polar plot illustrates the angular ranges for the four distinct coordination patterns defined in the present study. The classification of the different angular intervals follows the method proposed by Needham et al. (2015). The gray notations around the circumference (e.g., D50-P50, D100-P0) quantify the percentage of dominancy between the distal (D) and proximal (P) joints.

Figure 3 provides a visual representation of the four coordination patterns defined in this study, where each colored zone in the polar plot corresponds to a distinct pattern.

For each condition, the mean coupling angle ( ${\bar{\gamma }}_{i}$ ) was calculated from the coupling angle data of each trial. Because these data are directional, circular statistics were employed. First, the mean cosine ( ${\bar{x}}_{i}$ ) and sine ( ${\bar{y}}_{i}$ ) were calculated from the coupling angles ( $\gamma_{i}$ ) across all n time points within a given phase (Equations 2, 3).

$${\bar{x}}_{i}=\frac{1}{n}\sum_{i=1}^{n}\cos\gamma_{i}$$ (2)

$${\bar{y}}_{i}=\frac{1}{n}\sum_{i=1}^{n}\sin\gamma_{i}$$ (3)

Subsequently, the resultant mean angle ( ${\bar{\gamma }}_{i}$ ) was determined using Equation 4, which ensures the final value lies between 0° and 360° (Needham et al., 2014).

$${\bar{\gamma }}_{i}=\left\{\begin{array}{ll} \mathrm{Atan}\left(\frac{{\bar{y}}_{i}}{{\bar{x}}_{i}}\right)\cdot \frac{180}{\pi } & x_{i}>0,y_{i}>0\\ \mathrm{Atan}\left(\frac{{\bar{y}}_{i}}{{\bar{x}}_{i}}\right)\cdot \frac{180}{\pi }+180 & x_{i}<0\\ \mathrm{Atan}\left(\frac{{\bar{y}}_{i}}{{\bar{x}}_{i}}\right)\cdot \frac{180}{\pi }+360 & x_{i}>0,y_{i}<0\\ 90 & x_{i}=0,y_{i}>0\\ -90 & x_{i}=0,y_{i}<0\\ undefined & x_{i}=0,y_{i}=0 \end{array}\right.$$ (4)

### 2.4 Statistical analysis

Given that the primary dependent variable in this study was the mean coupling angle, all statistical inferences were performed based on the principles of circular statistics. To comprehensively evaluate the effects of shooting distance, movement phase, and their interaction on the mean coupling angle, separate Bayesian circular mixed-effects models were fitted for each of the six bilateral joint couplings. These models were implemented in R (version 4.5.1; R Core Team, 2025) using the bpnreg package (Cremers and Klugkist, 2018). The model formula is as follows (Equation 5) where the mean coupling angle ( $MeanCouplingAngle_{rad}$ ) served as the circular dependent variable. Shooting distance ( Condition ) and movement phase ( Phase ) were treated as fixed effects, while participant ( Subject ID ) was included as a random intercept:

$$MeanCouplingAngle_{rad}∼\;Condition\;*\;Phase+\left(1\;|\;Subject\;ID\right)$$ (5)

Each model was fitted using four separate Markov Chain Monte Carlo (MCMC) chains, each running for 35,000 iterations after a 13,000-iteration burn-in period, with a thinning interval of 8. Model convergence was confirmed by ensuring that the potential scale reduction factor ( $\hat{R}$ ) for all parameters was below 1.01 and that the effective sample size (ESS) was sufficient, indicating stable and well-mixed chains. All analyses were performed using pre-set seeds to ensure full reproducibility.

We first examined the interaction effect between distance and phase, using P1 and the Loading Phase as the baseline for the analysis. The interaction effect was interpreted as how the phase difference (Jump vs. Loading) at a given shooting distance deviates from the phase difference observed at the baseline distance. For each joint coupling, the follow-up analysis was guided by the overall significance of the interaction effect; specifically, if evidence for an interaction was observed in any comparison condition for a given joint coupling, we proceeded to analyze the simple main effects for that entire joint coupling. This involved assessing the differences in mean coupling angle among the shooting distances, conducted separately within each of the two phases. Conversely, if no such evidence for an interaction was found across all comparison conditions for a joint coupling, we then examined the main effects of distance and phase independently. The calculation of these effects was derived from the model’s full posterior distribution. For each posterior draw, we obtained population-level marginal predicted angles (excluding subject-specific random effects). Pairwise differences for simple main effects were computed as shortest circular differences. For condition main effects, we first formed the circular mean of the jump and loading predictions within each condition before taking the circular difference between conditions. For the overall phase main effect, we circularly averaged predictions across conditions within each phase before taking the circular difference (Jump − Loading).

In all comparisons, these differences represent the deviation of a given condition from the established baseline. All hypothesis tests were evaluated based on the 95% Highest Posterior Density (HPD) interval of the posterior difference. If the 95% HPD interval for a difference did not contain zero, this was interpreted as sufficient evidence for an effect. All results are presented in units of degrees.

## 3 Results

### 3.1 Interaction effects between shooting distance and movement phase

Table 2 presents the mean coupling angles and their standard deviations for each joint coupling across all distance conditions and movement phases.

**TABLE 2:** Mean coupling angles for each joint coupling across the four shooting distances and two movement phases.

Joint couplings	P1 ( ° )	P2 ( ° )	P3 ( ° )	P4 ( ° )
Loading phase				
Hip-knee: R	23.46 ± 1.79	22.72 ± 1.72	22.35 ± 1.58	22.10 ± 2.08
L	23.39 ± 1.52	23.07 ± 1.40	23.13 ± 1.39	23.42 ± 1.72
Hip-ankle: R	46.50 ± 2.34	43.60 ± 2.25	43.76 ± 2.06	40.98 ± 2.53
L	42.28 ± 2.44	40.87 ± 2.57	41.27 ± 2.31	42.25 ± 2.78
Knee-ankle: R	70.02 ± 0.91	68.97 ± 0.91	69.66 ± 0.83	65.78 ± 2.09
L	65.01 ± 0.64	66.49 ± 0.83	66.63 ± 0.80	66.20 ± 0.98
Jump phase				
Hip-knee: R	214.73 ± 4.10	209.70 ± 3.52	207.84 ± 3.25	202.16 ± 3.66
L	208.65 ± 3.20	204.40 ± 2.88	202.58 ± 2.65	200.48 ± 2.80
Hip-ankle: R	226.62 ± 2.70	221.88 ± 2.72	222.53 ± 2.73	221.54 ± 3.47
L	225.94 ± 3.42	220.05 ± 3.59	217.30 ± 3.42	219.64 ± 3.75
Knee-ankle: R	232.41 ± 4.09	232.86 ± 4.52	234.10 ± 3.79	235.02 ± 4.17
L	238.59 ± 2.91	234.07 ± 3.68	233.25 ± 3.37	235.61 ± 3.10

Data are expressed as mean ± SD. P1, P2, P3, and P4 represent the shooting distances of 4.6 m, 5.8 m, 7.0 m, and 8.325 m, respectively. R stands for the right side and L for the left side.

To explore whether the association between distance and the mean coupling angle differed between phases, we examined the interaction effects. This involved comparing each distance condition to the baseline (P1) within each phase (baseline: Loading Phase) in the joint pair models. Table 3 displays the posterior estimates for these interaction effects.

**TABLE 3:** Interaction effects of shooting distance and movement phase on the mean coupling angle for the lower limb joint couplings.

Comparison	Mean differences ( ° )	95% HPD [LB, UB] ( ° )
R_Hip-knee		
P1 vs. P2	−4.29	[-9.31, 0.57]
P1 vs. P3	−5.78	[-10.86, −0.72]*
P1 vs. P4	−11.22	[-16.67, −5.95]*
R_Hip-ankle		
P1 vs. P2	−1.84	[-7.81, 4.08]
P1 vs. P3	−1.36	[-7.23, 4.51]
P1 vs. P4	0.44	[-6.48, 7.28]
R_Knee-ankle		
P1 vs. P2	1.51	[-3.94, 6.82]
P1 vs. P3	2.04	[-2.95, 6.83]
P1 vs. P4	6.85	[1.02, 12.76]*
L_Hip-knee		
P1 vs. P2	−3.93	[-9.27, 1.53]
P1 vs. P3	−5.82	[-11.21, −0.50]*
P1 vs. P4	−8.20	[-13.73, −2.75]*
L_Hip-ankle		
P1 vs. P2	−4.50	[-10.62, 1.76]
P1 vs. P3	−7.64	[-13.67, −1.65]*
P1 vs. P4	−6.27	[-12.63, 0.16]
L_Knee-ankle		
P1 vs. P2	−6.01	[-11.18, −0.87]*
P1 vs. P3	−6.97	[-11.75, −2.20]*
P1 vs. P4	−4.17	[-8.75, 0.51]

Values are mean differences in degrees [95% HPD, Interval]. * indicates that the 95% HPD, interval for the difference did not contain zero, which was our criterion for observing evidence of an effect. Baselines are Point 1 and the Loading Phase. P1, P2, P3, and P4 represent the shooting distances of 4.6 m, 5.8 m, 7.0 m, and 8.325 m, respectively. R stands for the right side and L for the left side.

Analysis of the interaction effects (baseline: P1, Loading Phase) revealed evidence of interactions in multiple lower limb joint couplings. In our sample, evidence for an interaction was observed in the Hip-Knee and Knee-Ankle couplings on the right side, and across all three measured couplings (Hip-Knee, Hip-Ankle, and Knee-Ankle) on the left side. This observation indicates a pattern where, in our sample, the effect of distance on coordination differed between phases (all statistical details are presented in Table 3).

Further analyses of the interaction effect were conducted with P2 and P3 as respective baselines for distance, alongside the Loading Phase as the baseline for phase. Evidence for an interaction was also found for the right Hip-Knee coupling at P2 vs. P4 (−6.93 ° , 95% HPD [-11.81 ° , −2.02 ° ]) and P3 vs. P4 (−5.44 ° , 95% HPD [-10.42 ° , −0.71 ° ]). Full details are provided in Supplementary Table S1.

### 3.2 Simple main effects for joint couplings with evidence of interactions

A simple main effects analysis was conducted to further explore the association between distance and mean coupling angle within the Loading Phase (Table 4) and Jump Phase (Table 5) for those joint couplings that showed evidence of interaction between distances and phases.

**TABLE 4:** Simple main effects of shooting distance on mean coupling angles during the Loading Phase.

Comparison	Mean difference ( ° )	95% HPD [LB,UB] ( ° )
R_Hip-knee		
P1 vs. P2	−0.74	[-2.83, 1.29]
P1 vs. P3	−1.11	[-3.14, 0.91]
P1 vs. P4	−1.36	[-3.74, 1.00]
R_Knee-ankle		
P1 vs. P2	−1.06	[-2.25, 0.13]
P1 vs. P3	−0.36	[-1.55, 0.76]
P1 vs. P4	−4.24	[-7.22, −1.31]*
L_Hip-knee		
P1 vs. P2	−0.32	[-2.63, 2.00]
P1 vs. P3	−0.26	[-2.57, 2.05]
P1 vs. P4	0.02	[-2.59, 2.62]
L_Hip-ankle		
P1 vs. P2	−1.40	[-4.98, 2.13]
P1 vs. P3	−1.00	[-4.30, 2.39]
P1 vs. P4	−0.03	[-3.80, 3.69]
L_Knee-ankle		
P1 vs. P2	1.48	[0.32, 2.68]*
P1 vs. P3	1.63	[0.47, 2.74]*
P1 vs. P4	1.19	[-0.21, 2.56]

Values are mean differences in degrees [95% HPD, Interval]. * indicates that the 95% HPD, interval for the difference did not contain zero, which was our criterion for observing evidence of an effect. P1, P2, P3, and P4 represent the shooting distances of 4.6 m, 5.8 m, 7.0 m, and 8.325 m, respectively. R stands for the right side and L for the left side.

**TABLE 5:** Simple main effects of shooting distance on mean coupling angles during the Jump Phase.

Comparison	Mean difference ( ° )	95% HPD [LB, UB]
R_Hip-knee		
P1 vs. P2	−5.03	[-9.63, −0.70]*
P1 vs. P3	−6.89	[-11.54, −2.52]*
P1 vs. P4	−12.58	[-17.43, −7.69]*
R_Knee-ankle		
P1 vs. P2	0.45	[-4.78, 5.69]
P1 vs. P3	1.68	[-3.02, 6.44]
P1 vs. P4	2.61	[-2.38, 7.66]
L_Hip-knee		
P1 vs. P2	−4.25	[-9.09, 0.60]
P1 vs. P3	−6.08	[-10.93, −1.33]*
P1 vs. P4	−8.17	[-13.26, −3.30]*
L_Hip-ankle		
P1 vs. P2	−5.90	[-10.91, −0.76]*
P1 vs. P3	−8.65	[-13.74, −3.79]*
P1 vs. P4	−6.30	[-11.51, −1.23]*
L_Knee-ankle		
P1 vs. P2	−4.52	[-9.51, 0.39]
P1 vs. P3	−5.34	[-10.07, −0.85]*
P1 vs. P4	−2.98	[-7.28,1.48]

Values are mean differences in degrees [95% HPD, Interval]. * indicates that the 95% HPD, interval for the difference did not contain zero, which was our criterion for observing evidence of an effect. P1, P2, P3, and P4 represent the shooting distances of 4.6 m, 5.8 m, 7.0 m, and 8.325 m, respectively. R stands for the right side and L for the left side.

Within the Loading Phase, patterns of change associated with increasing shooting distance were primarily observed in the mean coupling angles of Knee-Ankle couplings for both sides. Evidence of a difference was observed in the right Knee-Ankle coupling at P1 vs. P4 (−4.24 ° , 95% HPD [-7.22 ° , −1.31 ° ]), and also in the left Knee-Ankle coupling at P1 vs. P2 (1.48 ° , 95% HPD [0.32 ° , 2.68 ° ]) and P1 vs. P3 (1.63 ° , 95% HPD [0.47 ° , 2.74 ° ]).

In contrast, the analysis of data from the Jump Phase showed a more widespread association between coordination and shooting distance. A majority of comparisons against the baseline (P1) yielded 95% HPD intervals that did not contain zero, indicating a consistent pattern of coordination alteration across most joint couplings (see Table 5 for full details).

A further analysis was conducted using P2 and P3 as sequential baselines. During the Loading Phase, this analysis also revealed evidence of a difference in the right Knee-Ankle coupling for both the P2 vs. P4 (−3.19 ° , 95% HPD [-6.14 ° , −0.26 ° ]) and P3 vs. P4 (−3.88 ° , 95% HPD [-6.88 ° , −0.86 ° ]) comparisons. During the Jump Phase, evidence of differences was also noted in the right Hip-Knee coupling at P2 vs. P4 (−7.55 ° , 95% HPD [-11.87 ° , −3.30 ° ]) and P3 vs. P4 (−5.69 ° , 95% HPD [-9.48 ° , −1.62 ° ]). Full details are provided in Supplementary Table S2, 3.

### 3.3 Main effects for joint couplings without evidence of interactions

For the right Hip-Ankle coupling where no evidence of an interaction effect was previously observed, we proceeded to examine the main effect of distance (Table 6) and the overall main effect of phase (Table 7) independently.

**TABLE 6:** Main effect of distance on mean coupling angles for right Hip-Ankle coupling.

Comparison	Mean difference ( ° )	95% HPD [LB, UB] ( ° )
R_Hip-ankle		
P1 vs. P2	42.71	[-6.71, 177.87]
P1 vs. P3	39.84	[-6.23, 178.25]
P1 vs. P4	36.05	[-8.57, 176.63]
P2 vs. P3	−9.59	[-180.00, 178.87]
P2 vs. P4	25.72	[-178.47, 180.00]
P3 vs. P4	29.63	[-5.72, 180.00]

Values are mean differences in degrees [95% HPD, Interval]. * indicates that the 95% HPD, interval for the difference did not contain zero, which was our criterion for observing evidence of an effect. P1, P2, P3, and P4 represent the shooting distances of 4.6 m, 5.8 m, 7.0 m, and 8.325 m, respectively. R stands for the right side and L for the left side.

**TABLE 7:** Overall main effect of phase on mean coupling angles for right Hip-Ankle coupling.

Comparison	Mean difference ( ° )	95% HPD [LB, UB]
R_Hip-ankle	18.26	[-179.50, 179.99]

Values are mean differences in degrees [95% HPD, Interval]. * indicates that the 95% HPD, interval for the difference did not contain zero, which was our criterion for observing evidence of an effect. R stands for the right side.

Our analysis for the right Hip-Ankle coupling was inconclusive (Table 6; Table 7). The 95% HPD intervals not only contained zero but were also extremely wide, indicating high uncertainty. Therefore, the current data are insufficient to either support or reject an effect for this joint coupling.

## 4 Discussion

While the single-joint analysis common in previous research has highlighted key kinematic adjustments (Cabarkapa and Cabarkapa, 2022; França et al., 2022), such an approach may overlook how the motor system functions as an interconnected network to produce flexible and stable performance. Given that athletes may exhibit more optimized coordination patterns with increasing skill level (Hafer et al., 2019), analyzing the time-series data of inter-joint movements is crucial for revealing the underlying coordination structure of skilled performance (Davids et al., 2003). Indeed, this approach allowed the present exploratory study to identify several key coordination patterns. Primarily, we observed a pattern of increased Distal Dominancy coordination during the Jump Phase in most of the examined joint couplings. Alongside this primary pattern, we identified a noteworthy asymmetrical adjustment in the bilateral Knee-Ankle joints during the Loading Phase, where the right side showed enhanced Distal Dominancy while the left trended towards Proximal Dominancy.

In our sample, the most pronounced pattern of adaptation was observed during the Jump Phase. With increasing shooting distance, coordination in most joint couplings manifested as an enhanced dominancy of the distal joints (knee and ankle). This observation suggests this strategic adaption may be consistent with an optimization of the “kinetic chain”, a mechanism whose purpose, as a proximal-to-distal linked system, is to achieve maximal velocity at the terminal segment through the sequential transfer of forces and motions (Putnam, 1993; Oliver et al., 2018), a crucial adaptation for meeting the demands of long-range shooting. This proposed kinematic mechanism is consistent with findings at the neuromuscular control level; for instance, it has been demonstrated that with greater shooting distances, there is a significant increase in the synergistic contribution of knee and ankle extensor muscles (i.e., rectus femoris and lateral gastrocnemius) during the shot’s release phase (Fan et al., 2024). This consistency suggests that the coordination pattern observed in our study and the reported alterations in muscle activation could be two facets of the same underlying adaptive strategy, a premise that warrants rigorous testing in a future confirmatory study.

The observed pattern of increased Distal Dominancy suggests a potential link between this coordination strategy adaptation and specific training modalities. Since this pattern is concentrated in the lower limb extension that characterizes the Jump Phase, it can be hypothesized that exercises accentuating this movement would be particularly effective. For instance, Olympic weightlifting and its derivatives (such as jump shrugs) have been shown to develop the explosive power critical for this phase (Hackett et al., 2016; Kipp et al., 2021). Therefore, we speculate that training regimens that incorporate exercises such as jump shrugs might facilitate the specific coordination adaptations required for long-range shooting. However, longitudinal studies are needed to verify this.

The Loading Phase is critical for energy management via the Stretch-Shortening Cycle (SSC) mechanism (Seiberl et al., 2021). During the swift downward movement of this phase, the lower limb muscle-tendon complexes are stretched, which both stores significant elastic energy and activates the stretch reflex to augment neural drive for the subsequent propulsive phase (Seiberl et al., 2021). The patterns observed in our exploratory analysis may offer initial insights into how athletes modulate this SSC mechanism through coordination adjustments in long-range shooting. Specifically, the primary kinematic adjustments during the Loading Phase were observed in the Knee-Ankle couplings. On the right side, the Knee-Ankle couplings trended towards greater Distal Dominancy. In contrast, the left Knee-Ankle coupling exhibited an increase in Proximal Dominancy, highlighting a potential asymmetrical adjustment specifically within the bilateral Knee-Ankle coordination.

However, it remains controversial whether such limb asymmetries positively or negatively impact athletic performance. While some studies have found negative associations, the relationship between asymmetry and performance is generally considered complex and inconsistent (Bishop et al., 2018). In support of a more functional view, other researchers propose that asymmetry is a natural, ubiquitous, and functional feature of human movement that may depend on the specific task (Afonso et al., 2022). Given that all participants were right-handed shooters, a degree of functional asymmetry is expected. Therefore, given the exploratory nature of this study and the subtle magnitude of the observed effects, we propose a hypothesis that this divergence in coordination strategies may represent a functional adaptation to the specific demands of the basketball shooting motion. This premise warrants further investigation to deepen our understanding of elite motor strategies.

This study has certain limitations. First, the sample size was relatively small, and all participants were male, right-handed university athletes, which may limit the generalization of the findings to a broader population. Second, the analysis focused on lower limb coordination and did not include an analysis of upper limb coordination patterns, whereas the motion of the upper limb joints also contributes significantly to the shooting action. Finally, limitations inherent to the vector coding technique are acknowledged. The discretization of continuous coupling angles into specific categories simplifies the complex coordination dynamics and may separate biomechanically similar data points near bin boundaries. Therefore, future research incorporating kinetic analysis is recommended to further validate the functional implications of these coordination strategies. Additionally, future research should aim to validate these findings in larger and more diverse populations. Expanding the analysis to include the upper limbs would provide a more holistic understanding of the whole-body coordination strategies, particularly the kinetic chain linkage between the lower and upper extremities, used to adapt to varying shooting distances.

## 5 Conclusion

In conclusion, this exploratory study observed distinct patterns of adaptation in lower limb coordination as shooting distance increased. The most pronounced pattern was identified during the Jump Phase, where coordination in most couplings showed a trend towards a greater dominancy of the distal joints (knee and ankle). Moreover, an asymmetrical coordination pattern was observed in the Knee-Ankle coupling during the Loading Phase: the right side trended towards enhanced Distal Dominancy, whereas the left side trended towards Proximal Dominancy. Based on these observations, this paper proposes two hypotheses for future research: that this trend towards greater Distal Dominancy may reflect a functional optimization of the kinetic chain, and the observed asymmetry might be a functional adaptation to the basketball shooting motion. Future studies are required to test these hypotheses.

## Data Availability

The original contributions presented in the study are included in the article/Supplementary Material, further inquiries can be directed to the corresponding authors.
