# Supplementary material for: Lower-limb coordination adaptations to shooting distance in basketball: an exploratory angular velocity-based vector coding study
Source: Front Bioeng Biotechnol. 2026 Jan 5;13:1730129. doi: 10.3389/fbioe.2025.1730129 (PMC12813038; doi:10.3389/fbioe.2025.1730129)
Supplement: Supplementary file 1 [file Table1.docx]

Supplementary Table 1. Supplementary results for interaction effects between Shooting Distance and Movement Phase

| Comparison | Mean Differences ($^{\circ}$) | 95% HPD [LB, UB] ($^{\circ}$) |
| --- | --- | --- |
| **R_Hip-Knee** |  |  |
| P2 vs. P3 | -1.49 | [-5.93, 2.88] |
| P2 vs. P4 | -6.93 | [-11.81, -2.02]* |
| P3 vs. P4 | -5.44 | [-10.42, -0.71]* |
| **R_Hip-Ankle** |  |  |
| P2 vs. P3 | 0.49 | [-5.29, 6.35] |
| P2 vs. P4 | 2.28 | [-4.48, 9.06] |
| P3 vs. P4 | 1.79 | [-4.88, 8.57] |
| **R_Knee-Ankle** |  |  |
| P2 vs. P3 | 0.54 | [-4.86, 5.80] |
| P2 vs. P4 | 5.35 | [-0.65, 11.18] |
| P3 vs. P4 | 4.81 | [-1.24, 10.66] |
| **L_Hip-Knee** |  |  |
| P2 vs. P3 | -1.89 | [-6.73, 3.18] |
| P2 vs. P4 | -4.27 | [-9.53, 0.97] |
| P3 vs. P4 | -2.38 | [-7.56, 2.67] |
| **L_Hip-Ankle** |  |  |
| P2 vs. P3 | -3.15 | [-9.35, 2.90] |
| P2 vs. P4 | -1.78 | [-8.11, 4.76] |
| P3 vs. P4 | 1.37 | [-5.07, 7.60] |
| **L_Knee-Ankle** |  |  |
| P2 vs. P3 | -0.96 | [-6.10, 4.20] |
| P2 vs. P4 | 1.84 | [-3.25,6.97] |
| P3 vs. P4 | 2.79 | [-1.98, 7.55] |
