# Supplementary material for: Lower-limb coordination adaptations to shooting distance in basketball: an exploratory angular velocity-based vector coding study
Source: Front Bioeng Biotechnol. 2026 Jan 5;13:1730129. doi: 10.3389/fbioe.2025.1730129 (PMC12813038; doi:10.3389/fbioe.2025.1730129)
Supplement: Supplementary file 2 [file Table2.docx]

Supplementary Table 2. Supplementary simple main effects results for Loading Phase

| Comparison | Mean Differences ($^{\circ}$) | 95% HPD [LB, UB] ($^{\circ}$) |
| --- | --- | --- |
| **R_Hip-Knee** |  |  |
| P2 vs. P3 | -0.37 | [-2.33, 1.61] |
| P2 vs. P4 | -0.62 | [-3.00, 1.73] |
| P3 vs. P4 | -0.25 | [-2.63, 2.14] |
| **R_Knee-Ankle** |  |  |
| P2 vs. P3 | 0.69 | [-0.44, 1.84] |
| P2 vs. P4 | -3.19 | [-6.14, -0.26]* |
| P3 vs. P4 | -3.88 | [-6.88, -0.86]* |
| **L_Hip-Knee** |  |  |
| P2 vs. P3 | 0.06 | [-2.18, 2.26] |
| P2 vs. P4 | 0.35 | [-2.22, 2.88] |
| P3 vs. P4 | 0.28 | [-2.24, 2.84] |
| **L_Hip-Ankle** |  |  |
| P2 vs. P3 | 0.40 | [-3.00, 3.90] |
| P2 vs. P4 | 1.37 | [-2.38, 5.18] |
| P3 vs. P4 | 0.97 | [-2.69, 4.69] |
| **L_Knee-Ankle** |  |  |
| P2 vs. P3 | 0.14 | [-1.11, 1.40] |
| P2 vs. P4 | -0.29 | [-1.74, 1.09] |
| P3 vs. P4 | -0.43 | [-1.84, 0.96] |
