# Supplementary material for: Lower-limb coordination adaptations to shooting distance in basketball: an exploratory angular velocity-based vector coding study
Source: Front Bioeng Biotechnol. 2026 Jan 5;13:1730129. doi: 10.3389/fbioe.2025.1730129 (PMC12813038; doi:10.3389/fbioe.2025.1730129)
Supplement: Supplementary file 3 [file Table3.docx]

Supplementary Table 3. Supplementary simple main effects results for Jump Phase

| Comparison | Mean Differences ($^{\circ}$) | 95% HPD [LB, UB] ($^{\circ}$) |
| --- | --- | --- |
| **R_Hip-Knee** |  |  |
| P2 vs. P3 | -1.86 | [-5.80, 2.00] |
| P2 vs. P4 | -7.55 | [-11.87, -3.30]* |
| P3 vs. P4 | -5.69 | [-9.84, -1.62]* |
| **R_Knee-Ankle** |  |  |
| P2 vs. P3 | 1.23 | [-4.01, 6.33] |
| P2 vs. P4 | 2.16 | [-3.03, 7.62] |
| P3 vs. P4 | 0.93 | [-3.98, 5.71] |
| **L_Hip-Knee** |  |  |
| P2 vs. P3 | -1.82 | [-6.21, 2.68] |
| P2 vs. P4 | -3.92 | [-8.57, 0.69] |
| P3 vs. P4 | -2.09 | [-6.64, 2.28] |
| **L_Hip-Ankle** |  |  |
| P2 vs. P3 | -2.75 | [-7.85, 2.25] |
| P2 vs. P4 | -0.41 | [-5.56, 4.83] |
| P3 vs. P4 | 2.34 | [-2.74, 7.43] |
| **L_Knee-Ankle** |  |  |
| P2 vs. P3 | -0.82 | [-5.79, 4.19] |
| P2 vs. P4 | 1.55 | [-3.40, 6.51] |
| P3 vs. P4 | 2.36 | [-2.16, 7.03] |
